# Supplementary material for: Two-dimensional type-II Dirac fermions in layered oxides
Source: Nat Commun. 2018 Aug 14;9:3252. doi: 10.1038/s41467-018-05715-2 (PMC6092334; doi:10.1038/s41467-018-05715-2)
Supplement: Supplementary file 1 — Supplementary Information [file 41467_2018_5715_MOESM1_ESM.pdf]

Two-dimensional Type-II Dirac Fermions in Layered Oxides

Horio *et al.*

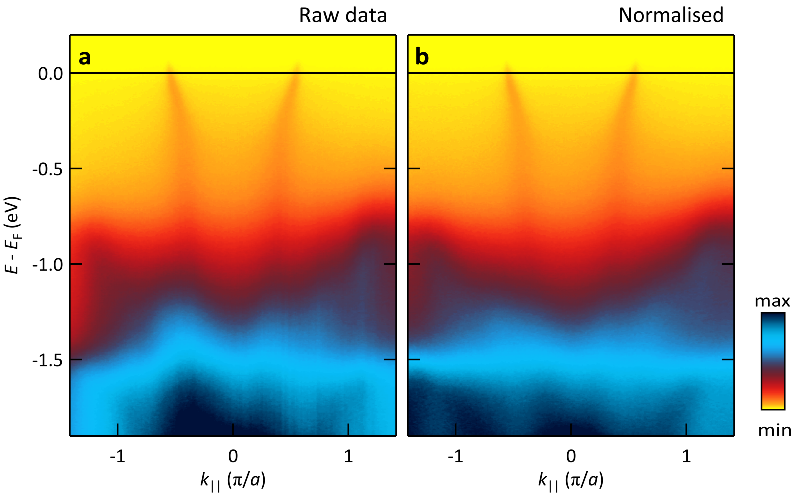

Supplementary Figure 1: **Channel normalisation.** (a,b) Raw and normalised energy distribution maps, respectively. For normalisation, the method (3) in Supplementary Note 1 has been employed. That is, each energy distribution curve has been normalised to its sum.

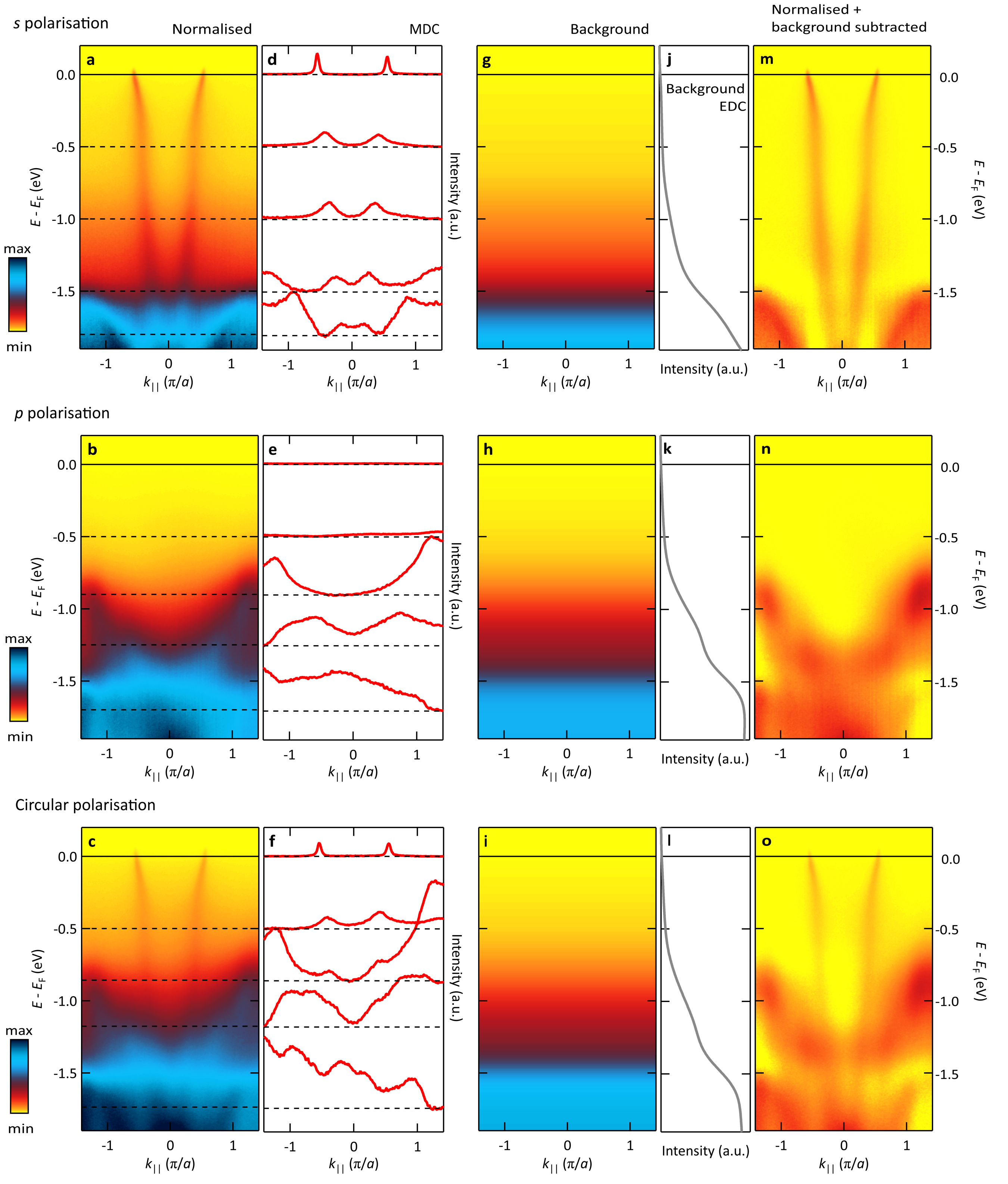

Supplementary Figure 2: **Background treatment.** Nodal spectra recorded on $La_{1.77}Sr_{0.23}CuO_4$ using *s*, *p*, and circular polarisation are shown in (a–c). (d–f) show MDCs at selected binding energies indicated by horizontal dashed lines in (a–c). An approximate background [dashed lines in (d–f)] is constructed averaging the five lowest observed intensities of each MDC and displayed in (g–i) and (j–l). The resulting background subtracted spectra are shown in (m–o).

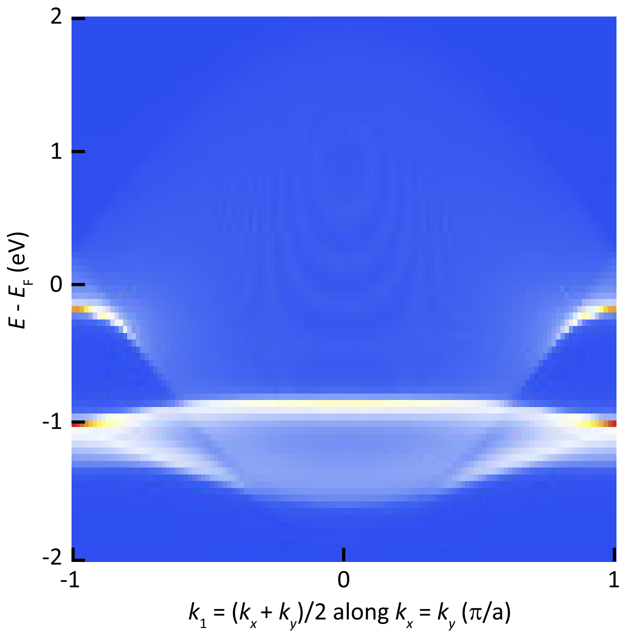

Supplementary Figure 3: **Calculated boundary modes.** Spectral function for Hamiltonian (1) of the main text in a slab geometry in which $k_1 = (k_x + k_y)/2$ and k_z are still good quantum numbers (*i.e.*, we consider layers stacked perpendicular to the nodal direction). The calculation is done for 100 layers and the spectral function of the top two layers ($k_z = 0$) is plotted. While the calculation does indicate some enhanced boundary spectral weight at $k_1 = \pi$, it is not connected in an obvious way to the location of the bulk type-II Dirac points.

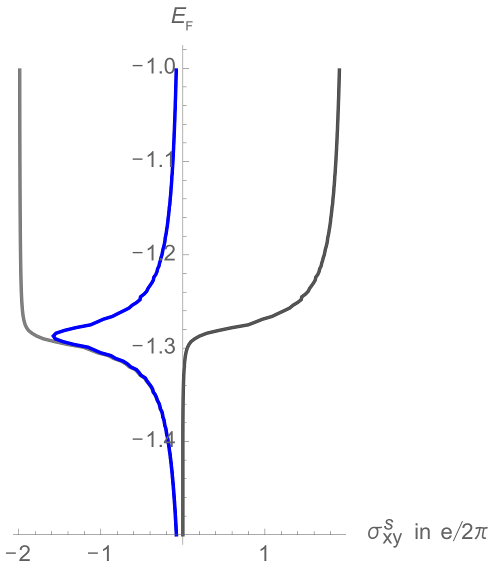

Supplementary Figure 4: **Spin Hall conductivity.** Contribution to the spin Hall conductivity σ^s_{xy} from each band of model (1)–(8) in the main text vs. Fermi level E_F (light grey and grey, respectively) as well as the total resulting spin Hall conductivity (dark blue).

Supplementary Note 1: Data treatment

Since the introduction of multi-channel photoemission electron analysers, normalisation to account for different channel efficiency's has been common practice. Virtually all published ARPES data has undergone this data treatment that allocate a multiplication factor to each of the measured energy distribution curves (EDCs). Different methodologies for this normalisation of efficiency has been developed. (1) EDCs are normalised to channel-integrated intensities measured on a poly-crystalline gold or copper reference. (2) The normalisation profile is extracted from the unoccupied side of the sample spectra. (3) Each energy distribution curve is normalised to its sum. For this work, we used the latter mentioned methodology. The effect of this normalisation is shown in Supplementary Fig. 1.

All measured ARPES spectra are composed of signal and background[1]. Here, we are not distinguishing between intrinsic and extrinsic background[1]. Both contributions (signal and background) vary as a function of binding energy and constitute so-called EDCs. Near the Fermi level the signal is typically much larger than the background that hence play no significant role. For large binding energies, however, the background can be considerable. Variation in background might therefore be larger than that of the signal and hence mask the signal in a energy distribution map.

To improve visualisation of our data, subtraction of the background is useful. It is, however, difficult to determine exactly the momentum and binding-energy dependent background. Here we therefore use an approximate method that typically underestimate the background for some momenta. The background for a fixed binding energy is obtained by averaging the five lowest observed intensities along the momentum distribution curve (MDC) – see supplementary Fig. 2. In this fashion, the shape of MDC is not influenced by the background subtraction. In fact, the background subtraction is nothing else than removing a growing offset from the MDCs. The procedure for the background construction and subtraction is demonstrated in Supplementary Fig. 2 for data obtained with different light polarisations.

Supplementary Note 2: Spin Hall conductivity

In this section, we compute the effect of spin-orbit coupling on the two-dimensional type-II Dirac fermions. Spin-orbit coupling lifts the degeneracy of the Dirac points, i.e., it induces a mass term. However, since the Dirac fermions are of type II, the resulting band structure is not insulating for any value of the Fermi energy (at least for small spin-orbit coupling). We consider the case where the mirror symmetry that sends $z \to -z$ is unbroken, so that z-component of spin is a good quantum number and the spin-current for polarisation in z-direction is conserved.

With the band structure not being insulating, we cannot expect the quantised spin-Hall conductivity for any value of the chemical potential. Still, as we show below, a substantial spin-Hall conductivity arises for Fermi energies near the energy of the – now massive – type-II Dirac nodes. In addition, spin-orbit coupling endows the two bands with opposite non-vanishing spin-Chern numbers C_s.

We define the spin Chern number $C_{\mathrm{s},n}$ of band n as in Ref. 2 as

$$C_{\mathrm{s},n} \equiv \sum_{\alpha=\uparrow,\downarrow} \alpha \frac{\mathrm{i}}{4\pi} \int_{\mathrm{BZ}} \mathrm{d}^2\mathbf{k} \left[\left\langle \frac{\partial u_{\mathbf{k},n,\alpha}}{\partial k_x} \middle| \frac{\partial u_{\mathbf{k},n,\alpha}}{\partial k_y} \right\rangle - \left\langle \frac{\partial u_{\mathbf{k},n,\alpha}}{\partial k_y} \middle| \frac{\partial u_{\mathbf{k},n,\alpha}}{\partial k_x} \right\rangle \right], \tag{1}$$

where $|u_{\mathbf{k},n,\alpha}\rangle$ is the Bloch state at momentum $\mathbf{k}$ with spin $\alpha = \uparrow, \downarrow$ in band n.

The spin-Hall conductivity, in contrast, is given by the above integral weighted with the Fermi distribution function[3]

$$\sigma^{\mathrm{s}}_{xy}(E_\mathrm{F}) = \frac{e}{2\pi} \sum_{\alpha=\uparrow,\downarrow} \sum_{n} \alpha \frac{\mathrm{i}}{4\pi} \int_{\mathrm{BZ}} \mathrm{d}^2\mathbf{k}\Theta(E_\mathrm{F} - E_{\mathbf{k},n}) \left[\left\langle \frac{\partial u_{\mathbf{k},n,\alpha}}{\partial k_x} \middle| \frac{\partial u_{\mathbf{k},n,\alpha}}{\partial k_y} \right\rangle - \left\langle \frac{\partial u_{\mathbf{k},n,\alpha}}{\partial k_y} \middle| \frac{\partial u_{\mathbf{k},n,\alpha}}{\partial k_x} \right\rangle \right], \tag{2}$$

where $E_{\mathbf{k},n}$ is the energy dispersion of band n. We have taken the zero temperature limit in which the Fermi distribution reduces to the Heavyside theta function Θ.

We evaluated $\sigma^{\mathrm{s}}_{xy}(E_\mathrm{F})$ as well as the contributions from each band $n = 1, 2$ for the model (1)–(8) in the main text with the parameters given below Eq. (9) as well as a spin-orbit coupling term given in Eq. (9) in the main text with $\lambda = 10$ meV. The result is plotted as a function of the Fermi level E_F in Supplementary Fig. 4 and indicates the estimated spin Hall response, which is unquantised but nevertheless results from the topologically non-trivial nature of the bands for finite spin-orbit coupling. For large E_F, the contributions from the two bands tend to the quantised

values $\pm e/\pi$, reflecting their nontrivial spin-Chern number.

[1] Kaminski, A. *et al.* Identifying the background signal in angle-resolved photoemission spectra of high-temperature cuprate superconductors. *Phys. Rev. B* **69**, 212509 (2004).

[2] Sheng, D. N. *et al.* Quantum Spin-Hall Effect and Topologically Invariant Chern Numbers. *Phys. Rev. Lett.* **97**, 036808 (2006).

[3] Matthes, L. *et al.* Intrinsic spin hall conductivity in one-, two-, and three-dimensional trivial and topological systems. *Phys. Rev. B* **94**, 085410 (2016).
